# COVID-19 prevention and control measures and experiences during the 14th National Games of China: a qualitative interview study

**DOI:** 10.3389/fpubh.2023.1271615

**Published:** 2024-01-22

**Authors:** Nan Li, Shike Hou, Yongzhong Zhang, Lulu Yao, Tiantian Li

**Affiliations:** ^1^College of Management and Economics, Tianjin University, Tianjin, China; ^2^Institute of Disaster and Emergency Medicine, Tianjin University, Tianjin, China

**Keywords:** prevention and control measures, public health preparedness, COVID-19 pandemic, 14th National Games, mass gatherings

## Abstract

**Background:**

The 14th National Games was the first sporting mass gathering to be held in China in the context of the COVID-19 pandemic. It may increase the risk of severe acute respiratory syndrome coronavirus 2 transmission. In order to reduce the spread of the virus during the 14th National Games, the Chinese government took a series of public health measures, and ultimately no confirmed cases were found in the 14th National Games venues.

**Objective:**

This study aimed to discuss preventive and control measures used to respond to the COVID-19 pandemic during the 14th National Games.

**Methods:**

Five experts were selected for this study using a snowball sampling method, and semistructured and in-depth interviews were conducted. Based on grounded theory, the transcriptions were analysed and coded using Nvivo 12 software.

**Results:**

A theoretical model of the COVID-19 prevention and control measures at the 14th National Games of China was constructed. The model contains seven main components: the health risks of mass gatherings, crowd management, emergency medical care, allocation of emergency medical resources, pandemic emergency drills, the pandemic prevention and control management platform (Quanyuntong app), and emergency response plans.

**Conclusion:**

The study showed that the deployment of emergency medical resources was the most important for mass gatherings. This study not only expanded the applications of grounded theory but also serves as a reference for future scholars when conducting more in-depth empirical studies on public health countermeasures for mass gatherings and can inform organizers when holding mass gatherings.

## 1 Introduction

Since the emergence of the coronavirus disease 2019 (COVID-19) pandemic impacted, it had a great impact on people all over the world and this disease poses a substantial threat to global public health during mass gatherings (1). During the COVID-19 pandemic, prolonged or close contact with an infected person can lead to transmission of the virus, highlighting the roles of mass gatherings, mass migration and other mass aggregations of people in crowded spaces (e.g., cruise ships) in the emergence, persistence, and spread of novel pathogens, under which mass gatherings pose a severe threat to global health security (2, 3).

The World Health Organization (WHO) defines mass gatherings as “events attended by enough people to strain the planning and response resources of a community, state or nation” (4, 5). For example, sporting events, carnivals, political rallies, and religious activities (e.g., pilgrimages) are all mass gatherings (6). The National Games of the People’s Republic of China is a comprehensive event with the greatest scale, highest competition, and the highest participation among mass gatherings in China (7), which is held every 4 years.

It encompasses 35 large events and 410 small events, with 37 sports delegations (8). The finals of the 14th National Games involved more than 12,000 athletes, 6,000 delegation officials, and 4,200 technical officials participating in the games, more than 5,300 organizing committee staff and 15,000 volunteers involved in service and security work, and more than 1,500 journalists covering the 14th National Games (9).

Although sports, religion, politics, and other types of mass gatherings may increase the risk of contracting COVID-19, this social activity also is a source of confidence and happiness; thus, people hope to resume such events as soon as possible (10–12). The 14th National Games was the first sporting mass gathering held in China during the COVID-19 pandemic.

During the COVID-19 pandemic, most governments responded with the suspension or cancellation of mass gatherings and implementation of other nonpharmaceutical measures (13). For example, the 2020 Umrah pilgrimage was suspended, and the 2020 Hajj was strictly limited to only 1,000 domestic pilgrims, excluding persons aged ≥65 years or those with chronic diseases (14). In Senegal, the Grand Magic Festival of Touba took place on 6 October 2020 and attracted millions of participants from Senegal and beyond (15). A series of precautionary measures, including the mandatory wearing of masks, closed-loop management, and the nucleic acid strategy, the vaccination strategy were implemented, and these mass gatherings were successfully concluded without major public health incidents (16); These examples related to mass gatherings provided lessons and experiences for the planning of the 14th National Games of China.

Because of the impact of the COVID-19 epidemic, there were more difficulties and challenges than the 13th National Games. From July to September 2021, due to virus mutations, the COVID-19 epidemic rebounded in many places in China, several regions in China experienced a wave of local outbreaks, such as Jiangsu Province, Heilongjiang Province, and Fujian Province. The outbreak was identified as the Delta variant. Due to the delta variant, COVID-19 rebounded around the world. The General Administration of Sport of China postponed the National Games, which were originally scheduled from August to September, 2021 (17). Experts recruited by associated governmental departments drew up guidelines and programs for prevention and control of COVID-19 and spread of infectious disease, emergency medical care, and health administration. Fortunately, there were no confirmed cases of COVID-19 at the 14th National Games venue (18). Given its success and the scarcity of literature in this field, the current study was conducted to understand the strengths and weaknesses of public health countermeasures during the 14th National Games to provide recommendations for public health preparedness of future mass gatherings in the context of a pandemic.

## 2 Methods

### 2.1 Study design

This exploratory qualitative study consisted of a literature search and semistructured interviews used to obtain in-depth information and experiences on the prevention and control of COVID-19 during the 14th National Games.

### 2.2 Population

To ensure a diverse sample, participants were selected through purposive sampling and snowballing from 14th National Games stakeholders.

Identified stakeholders were contacted by phone or email and invited to participate in the study. Event organizers and healthcare staff willing to provide medical services for the 14th National Games during the COVID-19 pandemic participated in this study.

### 2.3 Data collection

After review of the relevant literature, a guide for interview questions was developed to explore the interview process and validated by experts in the field of health emergencies and our lead team members. To obtain the most information from participants, all interview questions were open-ended. The interview question guide covered a wide range of aspects related to prevention and control measures and preparation for COVID-19. The interviews were conducted in Chinese and then translated into English.

### 2.4 Theoretical saturation test

This study employed the grounded theory approach, which requires researchers to continuously collect and analyze interview texts, and to code them until reaching data saturation, so that a comprehensive and concise theory can be developed based on the data. To confirm the potential emergence of new categories and concepts, after completing the interview with the first participant, we went on to interview the next respondent and analyze the text, resulting in the identification of indeed new categories and concepts. By analogy, when we analyze the text of the interviews and find no new information, this indicates data saturation. Furthermore, we provided the coded theoretical model to the interviewees, who confirmed that the model accurately reflected the actual situation and required no additional categories. Considering the clarity and robustness of the extracted main categories, initial categories, and relationship descriptions, we then stop collecting new data.

### 2.5 Data analysis

**Figure 1:**
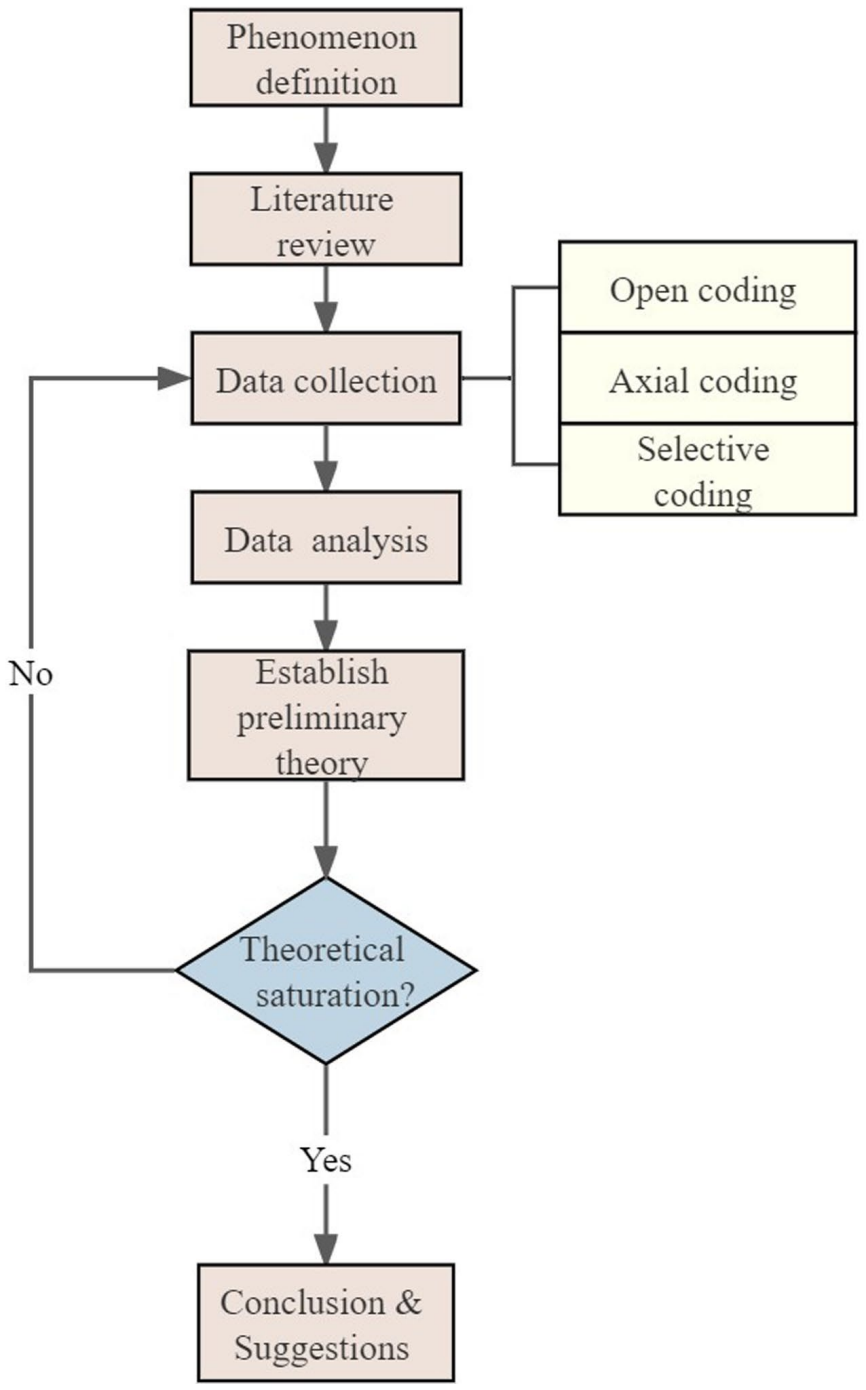
Flow chart of grounded theory analysis.

Based on grounded theory, developed by Glaser and Strauss (19) and Yue et al. (20), this study aimed to explore pandemic prevention and control measures. Grounded theory mainly includes the following steps: open coding, axial coding and selective coding. The specific process is shown in Figure 1.

The recorded interviews were transcribed verbatim, and the results were imported into Nvivo (version 12) software for analysis. The analysis included identifying, coding, and summarizing concepts and themes. Thematic analysis was performed on the transcripts by two researchers. This study recorded subtheme information that was repeatedly mentioned by some interviewees and that corresponded to previously reported information. Then, the researchers classified this information into different themes based on professional knowledge.

### 2.6 Ethical considerations

At the beginning of the interview, the interviewer introduced the research purpose, study background, and the definition of relevant terms. The interviewer sought informed consent from each participant. Their responses were anonymized to ensure that the interview contents were used only for academic research. The research protocol was reviewed and approved by the Tianjin University Ethics Committee (TJUE-2023-199).

## 3 Results

### 3.1 Characteristics of study participants

A total of eight respondents were invited through purposive sampling and snowballing. When two members of the research team analysed the text of the sixth interview, no new information was found, they were confident that data saturation had been reached. Therefore, a total of five interviewees were included in this study. The characteristics of the five respondents are described in Table 1. The interviews were conducted through Tencent Meeting software, and the average interview duration was 40 min (minimum: 20 min, maximum: 60.48 min). All participants were working in the healthcare department in Shaanxi Province. Details of the participants are provided in Table 1.

**Table 1:** Sociodemographic characteristics of participants.

ID	Sex	Age (years)	Work experience (years)	Highest degree	Service function	Occupation
1	Male	55	31	Bachelor’s	Decision-making and monitoring	Policymaker
2	Male	44	17	Master’s	Decision-making and monitoring	Policymaker
3	Male	37	13	Bachelor’s	Emergency medical treatment	Doctor
4	Male	37	11	Master’s	Emergency medical treatment	Doctor
5	Female	37	16	Bachelor’s	Front line pandemic prevention and control	Doctor

### 3.2 Open coding

Open coding is a basic coding stage that aims to identify phenomena, define concepts and refine categories (21). Based on the grounded theory principle of “localization”, the names of the open coding categories were developed based solely on the original text from interviews (22). A total of 47 tertiary nodes were obtained by combining, validating and coding the content of the five interview texts. The tertiary nodes at the bottom of the hierarchy involved the most detailed responses.

### 3.3 Axial coding

Axial coding occurs after open coding, according to Corbin and Strauss (23) paradigm model; combined with related theories, this step involves regrouping concepts and discovering and establishing organic links between conceptual categories (20). With the assistance of Nvivo 12 software, the 47 tertiary nodes of the interview text were summarized into 24 secondary nodes, which are located in the middle of the hierarchy.

### 3.4 Selective coding

Selective coding is based on spindle coding and involves identifying the core categories and establishing the relationship between the core categories and other categories (21). By further aggregating and integrating the 24 secondary nodes, 7 primary nodes were obtained: the health risks of mass gatherings, emergency response plans, COVID-19 emergency drills, crowd management, emergency medical care, allocation of emergency healthcare resources, and the COVID-19 pandemic prevention and control management platform (Quanyuntong app).

**Figure 2:**
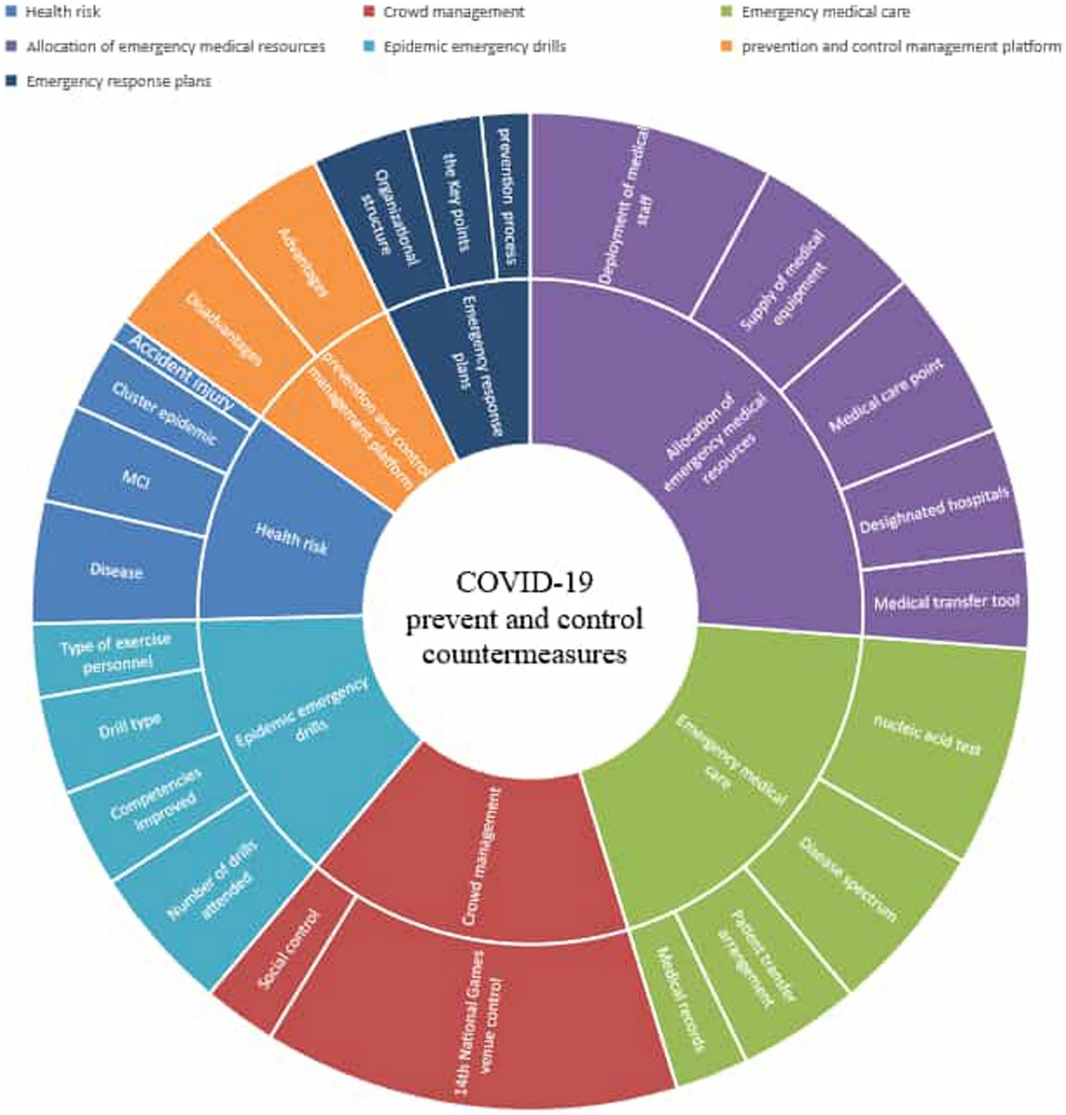
The structural model for the prevention and control of COVID-19 at the 14th National Games of China.

The coding results yielded a total of 126 coded reference points in this study. We found that the node of emergency medical resource deployment had the most reference points of all primary nodes and received the most attention, with the largest number of references (33), as shown in Figure 2. According to the number of coded reference points, the node of emergency medical resource deployment included 5 secondary nodes: designated hospitals, medical care points, medical transfer tools, supply of medical equipment, and deployment of medical staff. These secondary nodes included 66 reference points, accounting for 23.3% of the total number of reference points. Table 2 shows coding statistics of interview text.

**Table 2:** Coding statistics of interview text.

Primary nodes	Secondary nodes	Tertiary nodes
Allocation of emergency medical resources	Deployment of medical staff	Specialization of medical personnel, number of medical personnel.
	Designated hospitals	Number of designated hospitals, location of designated hospitals, type of designated hospitals.
	Medical care point	Number of medical care point, location of medical care point, equipment for medical care point.
	Medical transfer tool	Types of medical transfer tool, number of medical transfer tool
	Supply of medical equipment	Types of medical equipment
Emergency medical care	Nucleic acid test	Frequency of nucleic acid test, results of nucleic acid test
	Medical records	Approach to medical records (paper register or system entry).
	Patient transfer arrangement	Time spent in transit, transfer to destination hospital.
	Disease spectrum	Types of disease, trends in disease incidence.
Crowd management	Social control	Tourist attraction control, airport control.
	14th National Games venue control	Control of seating capacity, control of access to the venue.
Epidemic emergency drills	Drill type	Communication of information, process of critical incident response.
	Number of drills attended	Precomputation drills, simulation drills, on-site drills.
	Competencies improved	Coordination and communication skills, emergency response skills.
	Type of exercise personnel	Security, paramedics, other staff on site.
Health risk	Cluster epidemic	Food poisoning, infectious diseases.
	MCI	Factors influencing the occurrence of MCI
	Disease	Myocardial infarction, cardiac arrest
	Accident injury	Types of accidental injuries, number of accidental injuries
Prevention and control management platform	Advantages	Easy to operate, avoid cross infection
	Disadvantages	High workload, hard audit
Emergency response plans	Prevention process	Remote control, bubble strategy, pipeline transportation.
	Organizational structure	Three-tier organizational structure, Responsible for at each level of organization
	The Key points	Key technologies for epidemic prevention and control, Important factors in the absence of confirmed cases at the venue.

Figure 2 shows the structural model for the prevention and control response to the COVID-19 pandemic. The model is shown as a ring structure, with the centre representing the theme of the model. The multilayered ring displays the specific hierarchy of COVID-19 pandemic prevention and control responses, and the division of nodes within each ring layer reflects the dimensional categories. According to the model, the pandemic prevention and control response consisted of 7 primary nodes and 24 secondary nodes. The vertical structure of each sector in the ring reflects the hierarchical relationship of responses. The area of each sector in the ring represents the number of encoded reference points; the larger the sector area is, the more reference points it has. The number of encoded reference points reflects the number of documents that support that node.

Among the primary nodes, the medical resource deployment node had the most reference points and received the most attention. For example, some participants said in the interviews:

We have established medical care points for each competition, with the number of medical care point depending on the scale of the game. Each medical station is staffed by either one doctor and one nurse or two doctors and two nurses. The majority of medical staff providing support are from the Emergency Department. Additionally, we allocated doctors of different specialties according to the type of each game (participant 1).

In the marathon, at least one medical care point is set up every two kilometers, as the half-marathon is 21 kilometers and the full is more than 20 points. We are usually equipped with experienced, at least 4 experienced doctors. They come from cardiovascular, respiratory critical care, emergency medicine, orthopedics, trauma surgery doctors and will be trained in advance. The end of the half-marathon is particularly important, the bigger the medical care points, the better. In addition, the last 5 kilometers from 17–21 can arrange more medical care points, but also equipped with comprehensive strength of the hospital as a designated hospital. Because in the last 17 to 20 kilometers, the human body reached its limit, which is also the most likely to sudden heart attack (participant 2).

Emergency medical care, crowd management, and pandemic emergency drills also had many reference points and thus played an important role in the model. One participant commented:

I have participated in 10 emergency drills. The drills mainly involve communication with other departments, internal communication within the medical team, and cooperation with security personnel, event organizers, and referees. In terms of emergency medical treatment, we would select at least three types of hospitals as designated hospitals. Firstly, we would choose a hospital that is close in proximity to ensure prompt access to medical services. Secondly, we would select a hospital with a strong orthopedic or trauma department to address emergencies in those areas. Lastly, we would opt for a comprehensive tertiary-level hospital with strong overall capabilities to provide high-level emergency care (participant 3).

I’ve participated in drills related to entrances and exits and medical evacuation before the 14th Games. For example, if we find someone injured at the venue, what do we do? Which point has a person with fever that we need to transfer, how do we do it. How much time is needed to transfer people to the medical care point or the designated hospital, and how do we do it (participant 5).

Although there were relatively few reference points for the Quanyuntong app and plan, these aspects were an integral part of the overall response. Of all the secondary nodes, the 14th National Games venue control node had the most reference points, which indicates that control of the venue was very important. One participant commented:

In our plan, we have established a three-tier organizational structure. The first tier is the 14th National Games Organizing Committee, which is responsible for overseeing the entire event. At the municipal level, we have set up the second tier, known as the Executive Committee, to handle the operational aspects of the Games. Additionally, at the district and county level, we have established the third tier, known as the Competition Committee, to manage the specific competitions (participant 2).

We have implemented closed-loop management (also called the bubble policy) for all participants of the 14th National Games, with groups coming in and out to avoid contact with the outside world and reduce the risk of infection. At the same time, the maximum capacity allowed in the auditorium is 50% to prevent crowdedness and causing cross-contamination (participant 4).

**Figure 3:**
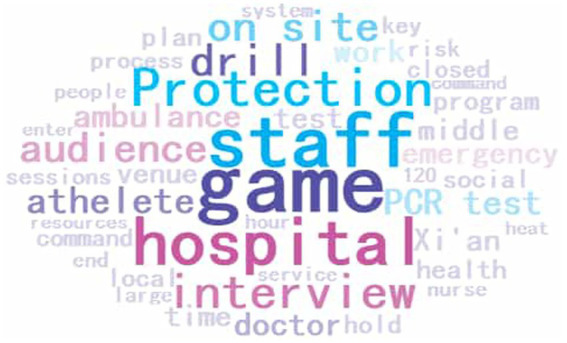
Word cloud for the COVID-19 pandemic control measures of the 14th National Games.

Nvivo 12 generates word clouds, which are visual representations of unstructured text data (24), as shown in Figure 3. The frequency with which these words appear determines their size and colour in the word cloud. In this study, the number of displayed phrases was set at 100, and the maximum length of displayed phrases was 2 words. In Figure 3, the word cloud shows a total of 108 words. The largest words, such as hospital, staff, and COVID-19 pandemic, were most frequently used in interview texts.

## 4 Discussion

Despite the timing of the mass gathering (i.e., during the COVID-19 pandemic), no confirmed cases of COVID-19 were recorded at the 14th National Games venue. This study collected the experiences and measures implemented by organizers to prevent the spread of COVID-19 by conducting in-depth interviews with five experts. The interview texts were then analysed in a bottom-up approach based on grounded theory, leading to the development of a theoretical model of the COVID-19 pandemic response. The theoretical model in Figure 2 revealed seven themes that occurred in interviews regarding the COVID-19 prevention and control measures implemented at the 14th National Games: the health risks of mass gatherings, crowd management, emergency medical care, allocation of emergency medical resources, pandemic emergency drills, the pandemic prevention and control management platform, and emergency response plans.

Notably, the allocation of emergency medical resources was vital. During the COVID-19 pandemic, healthcare workers were under tremendous pressure to serve as social leaders and guide the public. Therefore, the deployment and management of healthcare workers should be the first topic that policymakers consider to ensure that they can better provide care to patients, which is consistent with previous studies (25). Furthermore, many emergency medical teams in China are composed of staff drawn from emergency departments and other related departments on a temporary basis, and the lack of clear criteria for performance evaluation of this staff is a challenge that will need to be addressed in the future. Many of the experts reported that the 14th National Games had implemented differentiated allocation of medical resources, for example, directing thoracic surgery and cardiovascular resources to the marathon and directing orthopedic resources to some ball games.

Providing training for emergency medical service providers prior to mass gatherings is a practical measure to ensure disaster preparedness. To enhance emergency response capacity, authorities conducted several exercises regarding the prevention and control of COVID-19 in Shaanxi Province. The training drills mainly involved precompetition drills, simulation drills and on-site drills. In the preplanning phase, the authorities examined the following three scenarios. (1) For close contacts of positive cases, the following measures were taken: timely transfer to centralized quarantine, investigation, and control of risk by specific personnel, monitoring, and disinfection. (2) For people who tested positive for COVID-19, the following measures were taken: the emergency command system was activated, a report was composed, information was transmitted in a timely manner, high-risk groups were identified, on-site quarantine measures were implemented, an epidemiological investigation was conducted, and disinfection was performed. (3) For dispersed confirmed cases or asymptomatic patients, the following measures were taken: the emergency command system was activated, emergency medical care was administered, an epidemiological investigation was performed, close contacts were identified and controlled, prevention and control areas were delineated, risk assessment was performed, active surveillance was performed, PCR testing was conducted, information was disseminated, and public opinions were monitored.

The 14th National Games epidemic prevention and control management platform is a smartphone app called Quanyuntong, which helped to prevent any confirmed cases from being found at the 14th National Games venue. The platform was divided into four parts: (1) personal information containing the identification number and identity category; (2) three operation tools: QR code scanning, The 14th National Games code and the ticket bundle; (3) four modules: a PCR test (negative or positive), COVID-19 vaccine information (the first injection, the first few months), health check-in (body temperature), abnormal epidemic prevention; and (4) four functions: The QR code of the accompanying staff, audit records, help centres, and other services. Especially with the accompanying code, family members could help older adult individuals or children who did not have smartphones to fill in the information. If participants later tested positive for COVID-19 and needed to be traced, the event organizers could quickly identify the activity trajectory of confirmed cases, as well as their close contacts, based on information from the platform. This reduced the time needed to trace the outbreak and prevent the disease from spreading. This platform is more accurate and faster than manual tracking regarding close contact tracing, as it provided a limited list of potential close contacts rather than an exhaustive list of all contacts (2).

Nonpharmaceutical interventions such as crowd management were key to prevention measures at the 14th National Games; such interventions involved limiting the maximum number of attendees entering the venue to half of the venue’s capacity and compulsory mask wearing. Experts have reported that masks act as a physical barrier to prevent the spread of virus-carrying droplets from people, the evidence that masks reduce the spread of disease is consistent with the findings of a US study (26, 27). In addition, the experts reported that limiting the maximum number of spectators entering the Games venues to half the capacity of the venue, maintaining a physical distance of 1 metre among individuals, and reducing overcrowding effectively reduced the spread of the virus. Previous studies have also shown that keeping people at a physical distance of one metre or more from each other sharply reduces the risk of disease spread (28–30).

Closed-loop management (also called the bubble strategy) is a management approach that has been developed from the principles of integrated information systems, closed-loop systems, management control, and management closure (31). An integrated system was created at the National Village, contract hotels, training venues, other pre-listed destinations, and dedicated connecting transportation to separate participants from residents at the 14th National Games. To maintain the integrity of the bubble, they were transported between destinations using vehicles with specific signage and followed dedicated routes into other bubbles.

It can be effectively applied to the prevention and control of COVID-19 outbreaks. Strict closed-loop management was implemented once the athletes arrived in Shaanxi for the 14th National Games. After arriving in Xi’an, the athletes, technical officials, and media staff were transported in special buses using private channels to the National Village or reception hotel to ensure closed management. There was a small fever clinic and medical station within the National Village, and patients could also choose to seek medical treatment at nearby designated hospitals, which had green channels for athletes to use. Closed-loop management measures were used to avoid contact between athletes and outsiders, effectively reduce crowd sizes and wait times, and reduce the possibility of cross-infection (31). The experts noted that group entries and exits, pipeline transport, and bubble management were all types of closed management.

A three-layer testing strategy was implemented during the 14th National Games, including predeparture testing, postarrival testing, and daily PCR testing. All participants in the 14th National Games provided proof of PCR testing within 48 h prior to arrival in Shaanxi. The PCR test was performed at airports and railway stations before access to the closed-loop system. Then they need participants underwent daily PCR tests. The PCR test played an important role in preventing the spread and controlling the virus (32, 33). In addition to all precautionary measures and clear instructions, the exclusion of all individuals who tested positive prior to traveling to the 14th National Games was a factor contributing to the no confirmed cases at the 14th National Games venue.

This is a retrospective study, and the interviewees are all experienced experts or doctors who participated in the 14th National Games. Through semi-structured interviews, they were asked to recall their valuable experiences in epidemic prevention and control during their participation in the 14th National Games. Through summarizing the experiences and insights of the interviewees, its practical guidance for pandemic prevention and control. Although this is not a scientific study in the traditional sense, the practical experience and insights of the experts are important inspirations for developing more effective prevention and control measures.

## 5 Conclusion

The 14th National Games was the first sporting mass gathering held in China during the COVID-19 pandemic and achieved 0 confirmed cases at the venue. The success originated from the efforts of the government, the 14th National Games authorities, and emergency medical workers. This experience provided lessons for national and international health organizations regarding other mass gatherings; a set of public health countermeasures were implemented in the context of the COVID-19 pandemic. Nonpharmaceutical interventions were paramount in China; other countries worldwide implemented prevention and control measures similar to those in China, but these measures were not entirely applicable to all countries.

### 5.1 Limitations

The main limitation of this study was the use of Tencent Meeting software for interviews rather than conducting face-to-face interviews due to distance and the need to protect the privacy of the interviewees. Interviews that lack any visual aids, such as behaviours and body language, may affect the results of the study. In addition, due to the limitations of the study data and materials, quantitative research methods were not used in this study to validate the results. We plan to quantitively analyze the findings in the next study.

## Data availability statement

The raw data supporting the conclusions of this article will be made available by the authors, without undue reservation.

## Ethics statement

The studies involving humans were approved by Tianjin University Ethics Committee. The studies were conducted in accordance with the local legislation and institutional requirements. The participants provided their written informed consent to participate in this study.

## Author contributions

NL: Investigation, Methodology, Writing – original draft, Writing – review & editing, Formal analysis. YZ: Conceptualization, Funding acquisition, Supervision, Writing – review & editing. SH: Conceptualization, Funding acquisition, Supervision, Validation, Writing – review & editing. LY: Data curation, Formal analysis. TL: Data curation, Investigation.

## References

[1] Khan A Bieh KL El-Ganainy A Ghallab S Assiri A Jokhdar H. Estimating the COVID-19 risk during the hajj pilgrimage. J Travel Med. (2020) 27:taaa157. doi: 10.1093/jtm/taaa157, PMID: 32889536 PMC7499715

[2] Ryan BJ Coppola D Williams J Swienton R. COVID-19 contact tracing solutions for mass gatherings. Disaster Med Public Health Prep. (2021) 15:e1–7. doi: 10.1017/dmp.2020.241, PMID: 32660677 PMC7403747

[3] Zhu N Zhang D Wang W Li X Yang B Song J. China novel coronavirus investigating and research team. A novel coronavirus from patients with pneumonia in China. N Engl J Med. (2019, 2020) 382:727–33. doi: 10.1056/NEJMoa2001017 PMC7092803 31978945

[4] WHO. Public health for mass gatherings: key considerations. World Health Organisation. Available at: https://apps.who.int/iris/bitstream/handle/10665/162109/WHO_HSE_GCR_2015.5eng.pdf;jsessionid=20CDEF66BA427C7DB54004047BD811E4?sequence=1 (Accessed June 21, 2021).

[5] Khan AA Sabbagh AY Ranse J Molloy MS Ciottone GR. Mass gathering medicine in soccer leagues: a review and Cre-ation of the SALEM tool. Int J Environ Res Public Health. (2021) 18:9973. doi: 10.3390/ijerph18199973, PMID: 34639274 PMC8508246

[6] Memish ZA Stephens GM Steffen R Ahmed QA. Emergence of medicine for mass gatherings: lessons from the hajj. Lancet Infect Dis. (2012) 12:56–65. doi: 10.1016/S1473-3099(11)70337-1, PMID: 22192130 PMC7185826

[7] Fei C Jianjin C Nan T. Reflections on the 14th National Games in the light of the novel coronavirus outbreak. J Nanjing Sport Inst. (2020) 19:7–12. doi: 10.15877/j.cnki.nsin.2020.05.002

[8] Zhejiang province delegation formed for the 14th National Games (2021). Available at: http://www.zj.gov.cn/art/2021/9/10/art_1554467_59128638.html (Accessed October 1, 2021).

[9] Continuing olympic excitement perfect finish of national games. Available at: https://news.cctv.com/2021/09/28/ARTIBCzKncLyTfvJvSHGZ1Dg210928.shtml (Accessed June 22, 2022)

[10] Chan JEZ Lee A Lease C Spurrier N. Recommencement of sport leagues with spectators at the Adelaide oval during the COVID-19 pandemic: planning, experience, and impact of a globally unprecedented approach. Front Public Health. (2021) 9:676843. doi: 10.3389/fpubh.2021.676843, PMID: 34368052 PMC8345120

[11] Murakami M Yasutaka T Onishi M Naito W Shinohara N Okuda T. Living with COVID-19: mass gatherings and minimizing risk. QJM. (2021) 114:437–9. doi: 10.1093/qjmed/hcab163, PMID: 34109393 PMC8344524

[12] World Health Organization. Key planning recommendations for mass gatherings in the context of the current COVID-19 outbreak: Interim guidance. (2020). Available at: https://www.who.int/publications/i/item/10665-332235 (Accessed June 26, 2022).

[13] McCloskey B Zumla A Ippolito G Blumberg L Arbon P Cicero A. Mass gathering events and reducing further global spread of COVID-19: a political and public health dilemma. Lancet. (2020) 395:1096–9. doi: 10.1016/S0140-6736(20)30681-4, PMID: 32203693 PMC7138150

[14] Jokhdar H Khan A Asiri S Motair W Assiri A Alabdulaali M. COVID-19 mitigation plans during hajj 2020: a success story of zero cases. Health Secur. (2021) 19:133–9. doi: 10.1089/hs.2020.0144, PMID: 33264063 PMC8060722

[15] Sokhna C Goumballa N Hoang VT Bassene H Parola P Gautret P. The grand Magal of Touba was spared by the COVID-19 pandemic. Int J Infect Dis. (2021) 105:470–1. doi: 10.1016/j.ijid.2021.01.006, PMID: 33434665 PMC9183244

[16] Zumla A Azhar EI Shafi S Memish ZA. COVID-19 and the scaled-down 2020 hajj pilgrimage – decisive, logical and prudent decision making by Saudi authorities overcomes pre-hajj public health concerns. Int J Infect Dis. (2020) S1201–9712:30643–3. doi: 10.1016/j.ijid.2020.08.014 PMC7413862 32777586

[17] Notice on postponement of the 14th National Games (2021). General Administration of Sport of China. Available at: https://www.sport.gov.cn/qts/n4986/c23457050/content.html (Accessed May 12, 2022).

[18] Health Commission of Taizhou (2021). Shaanxi experience of "zero infection" in 14th National Games. Available at: https://www.sport.gov.cn/qts/n4986/c23457050/content.html (Accessed May18, 2022).

[19] Glaser BG Strauss AL. The discovery of grounded theory: Strategies for qualitative research. Chicago, IL: Aldine Transaction (1967).

[20] Yue W Yu L Yang Y. The occupational anxiety of teachers caused by China's “double reduction” policy-a study based on the grounded theory. Front Psychol. (2023) 14:1144565. doi: 10.3389/fpsyg.2023.1144565 37034957 PMC10074598

[21] Cui PL. Influencing factors and models of undergraduate course participation research based on grounded theory. J Cangzhou Norm Coll. (2022) 3:106–14. doi: 10.13834/j.cnki.czsfxyxb.2022.03.020

[22] Haobo ZH Lingxiao LI. Study on the influencing factors of college teachers’ job satisfaction – a qualitative analysis of Nvivo based on 48 papers. Mod Educ Manag. (2019) 11:69–73. doi: 10.16697/j.cnki.xdjygl.2019.11.012.2

[23] Corbin J Strauss A. Basics of qualitative research: techniques and procedures for developing grounded theory. Newbury Park, CA: Sage publications (1990).

[24] Sun H Zafar MZ Hasan N. Employing natural language processing as artificial intelligence for analyzing consumer opinion toward advertisement. Front Psychol. (2022) 31:856663. doi: 10.3389/fpsyg.2022.856663 PMC9234938 35769737

[25] Riedel B Horen SR Reynolds A Hamidian JA. Mental health disorders in nurses during the COVID-19 pandemic: implications and coping strategies. Front Public Health. (2021) 26:707358. doi: 10.3389/fpubh.2021.707358 PMC8575697 34765579

[26] Rader B White LF Burns MR Chen J Brilliant J Cohen J. Mask-wearing and control of SARS-CoV-2 transmission in the USA: a cross-sectional study. Lancet Digit Health. (2021) 3:e148–57. doi: 10.1016/S2589-7500(20)30293-4 33483277 PMC7817421

[27] Hou YJ Okuda K Edwards CE Martinez DR Asakura T Dinnon KH III. SARS-CoV-2 reverse genetics reveals a variable infection gradient in the respiratory tract. Cells. (2020) 182:429–446.e14. doi: 10.1016/j.cell.2020.05.042 PMC7250779 32526206

[28] World Health Organization. WHO official updates—Coronavirus disease. Geneva: World health organization (2019). 2020 p.

[29] Setti L Passarini F De Gennaro G Barbieri P Perrone MG Borelli M. Airborne transmission route of COVID-19: why 2 meters/6 feet of interpersonal distance could not be enough. Int J Environ Res Public Health. (2020) 17:2932. doi: 10.3390/ijerph17082932 32340347 PMC7215485

[30] Aghdam FB Sadeghi-Bazargani H Shahsavarinia K Jafari F Jahangiry L Gilani N. Investigating the COVID-19 related behaviors in the public transport system. Arch Public Health. (2021) 79:183. doi: 10.1186/s13690-021-00702-4 34674753 PMC8531038

[31] Bai DS Geng P Wang ZD Wang XL Xu GR Ye Q. Practice and expe-rience of regional medical center entrance linkage and closed-loop management under the wartime situation of the COVID-19 in China. Ann Transl Med. (2022) 10:112. doi: 10.21037/atm-22-61 35282098 PMC8848449

[32] Tian H Liu Y Li Y Wu CH Chen B MUG K. An investigation of transmission control measures during the first 50 days of the COVID-19 epi-demic in China. Science. (2020) 368:638–42. doi: 10.1126/science.abb6105 32234804 PMC7164389

[33] Abbott S Hellewell J Thompson RN Sherratt K Gibbs HP Bosse NI. Estimating the time-varying reproduction number of SARS-CoV-2 using national and subnational case counts. Wellcome Open Res. (2020) 5:112.

